# Genetic Exchange of Fimbrial Alleles Exemplifies the Adaptive Virulence Strategy of *Porphyromonas gingivalis*

**DOI:** 10.1371/journal.pone.0091696

**Published:** 2014-03-13

**Authors:** Jennifer E. Kerr, Jared R. Abramian, Doan-Hieu V. Dao, Todd W. Rigney, Jamie Fritz, Tan Pham, Isabel Gay, Kavitha Parthasarathy, Bing-yan Wang, Wenjian Zhang, Gena D. Tribble

**Affiliations:** 1 Department of Periodontics and Dental Hygiene, School of Dentistry, University of Texas Health Science Center at Houston, Houston, Texas, United States of America; 2 Department of Diagnostic and Biomedical Sciences, School of Dentistry, University of Texas Health Science Center at Houston, Houston, Texas, United States of America; University of Osnabrueck, Germany

## Abstract

*Porphyromonas gingivalis* is a gram–negative anaerobic bacterium, a member of the human oral microbiome, and a proposed “keystone” pathogen in the development of chronic periodontitis, an inflammatory disease of the gingiva. *P. gingivalis* is a genetically diverse species, and is able to exchange chromosomal DNA between strains by natural competence and conjugation. In this study, we investigate the role of horizontal DNA transfer as an adaptive process to modify behavior, using the major fimbriae as our model system, due to their critical role in mediating interactions with the host environment. We show that *P. gingivalis* is able to exchange fimbrial allele types I and IV into four distinct strain backgrounds via natural competence. In all recombinants, we detected a complete exchange of the entire *fimA* allele, and the rate of exchange varies between the different strain backgrounds. In addition, gene exchange within other regions of the fimbrial genetic locus was identified. To measure the biological implications of these allele swaps we compared three genotypes of *fimA* in an isogenic background, strain ATCC 33277. We demonstrate that exchange of fimbrial allele type results in profound phenotypic changes, including the quantity of fimbriae elaborated, membrane blebbing, auto-aggregation and other virulence-associated phenotypes. Replacement of the type I allele with either the type III or IV allele resulted in increased invasion of gingival fibroblast cells relative to the isogenic parent strain. While genetic variability is known to impact host-microbiome interactions, this is the first study to quantitatively assess the adaptive effect of exchanging genes within the pan genome cloud. This is significant as it presents a potential mechanism by which opportunistic pathogens may acquire the traits necessary to modify host-microbial interactions.

## Introduction

Complex communities of commensal microorganisms comprise the vast majority of the human body’s natural flora. Within these thousands of species are many that are also opportunistic pathogens, capable of causing disease in the event of a failure of host defenses. Further subdividing the opportunistic pathogens, there are a few species that colonize as part of the normal flora, but may evolve within the host habitat to adopt a more overtly pathogenic phenotype. Typically this adaptation process is thought to be driven by spontaneous mutation and horizontal DNA transfer events; with the increasing availability of strain-level genome sequences, identification and molecular characterization of these adaptive virulence events is becoming possible [1], [2]. Understanding how these opportunistic bacteria shift from commensal to pathogenic in the host environment will prove pivotal in combating these resourceful organisms in the future.

*Porphyromonas gingivalis* is a gram-negative anaerobe that preferentially inhabits the human oral cavity and is a key etiologic agent in the development of periodontal disease. This bacterium colonizes established multi-species plaque biofilms at the gingival margin and in the sub-gingival sulcus [3]–[5]. Residence within sub-gingival biofilms places *P. gingivalis* and co-resident commensal microorganisms in close proximity to the non-keratinized junctional epithelium, and in a healthy periodontium, the bacterial burden is restricted by circulating neutrophils and innate immune effectors. Unlike its commensal neighbors, *P. gingivalis* has multiple strategies for undermining the long-term effectiveness of host defenses, and has been shown in a recent landmark study to be a “keystone” pathogen, able to disrupt host-microbe homeostasis by molecular manipulation of select host protective mechanisms [6]. The most important immune dysregulation events appear to be inhibition of IL-8 secretion, subversion of the complement system, and Toll-like receptor antagonism [7]. Inhibition of these host defenses results in a significant increase in total bacterial burden in the sub-gingival sulcus, and enables *P. gingivalis* and other opportunistic pathogens within the oral flora to overcome mucosal defenses and stimulate the inflammatory destruction of the periodontal connective tissues [3], [8]. The outcome of tissue destruction is beneficial to *P. gingivalis*: creation of the periodontal pocket, an enlarged sub-gingival space that provides ideal conditions for propagation of this strictly anaerobic, proteolytic microbe.

*P. gingivalis* colonization and survival in the host are attributable to a number of persistence factors, including biofilm formation, production of capsule, secretion of proteases, and the presence of major and minor fimbriae [9]. The major fimbriae are multifunctional adhesins, and mediate adherence to and signaling within bacterial biofilms and host tissues. Beta-1 integrin has been identified as the host receptor on multiple cell types; binding of integrin by the major fimbriae activates a cascade of events that result in internalization of *P. gingivalis* into the host cell, localized suppression of cytokine production, inhibition of apoptotic signaling pathways, and interference with cell differentiation and mineralization [10]–[13]. Major fimbriae also interact with TLR2 receptors, cross-linking them with CXCR4 to block receptor signaling [14], making fimbriae mechanistically important for key events in immune dysregulation [15].

The major fimbriae of *P. gingivalis* are primarily composed of the structural protein subunit FimA, encoded by the *fimA* gene [16], [17]; additional components are encoded by downstream genes *fimB*, *fimC*, *fimD*, and *fimE*. The FimBCDE proteins are not well characterized, but FimCDE appear to be minor structural components of the fimbria involved in adhesion specificity, and FimB, although not essential for fimbria assembly, is involved in regulation of fimbrial length [18]–[20]. Expression of the *fimA* gene is tightly regulated by environmental signals, and involves the two-component signal transduction system *fimSR*, and a transcriptional regulator *fimX* [21], [22]. The promoter region also requires binding by FimA protein and gingipain proteases for maximum activity [23]–[25]. Trafficking of FimA to the outer membrane occurs via a lipoprotein intermediate, and cleavage of a leader peptide prior to fimbria polymerization at the cell surface [26], [27]. The presence of fimbria is important for efficient biofilm formation, interaction with other bacterial species, and adhesion to and invasion of host cells. After internalization into host cells, expression of major fimbria is strongly down-regulated [28]; mutants lacking fimbriae are less efficient at host cell invasion, but once internalized have improved survival rates over time [29].

*P. gingivalis* is a genetically diverse species, and frequent exchange of virulence alleles between strains has been predicted to occur as part of generating a variable pathogenic potential [30]–[32]. The *fimA* gene is among the best studied of the allelic virulence factors, and *P. gingivalis* strains are classified into six types based on divergent nucleotide sequences of the *fimA* gene (types I and Ib to V; [33]). Numerous studies have reported *fimA* types II and IV are more common in strains isolated from periodontal pockets, and types I and Ib are more common in strains found in a healthy gingival sulcus or refractory periodontitis [34]–[39] [40]. It is not uncommon to detect multiple *fimA* types transiently occupying the same sub-gingival sulcus, although one strain is generally dominant in numbers [35], [41]–[43].

In recent years the molecular mechanisms underlying *P. gingivalis* genetic variability have become clearer. *P. gingivalis* is naturally competent and releases extracellular DNA (eDNA) in mature biofilms [44]. This eDNA is assimilated into recipient cell genomes and is predicted to provide a major source of genetic material for horizontal gene exchange via transformation between *P. gingivalis* strains. In addition, some strains of *P. gingivalis* are capable of conjugal transfer of chromosomal DNA and conjugative transposons [45], [46]. The presence of mechanisms for exchange of DNA within the species likely allows shuffling of alleles within the pan-genome to create a “cloud” of strains, the fittest of which will survive and dominate in the host environment [47]–[49].

In the present study, we tested the ability of *P. gingivalis* strains to exchange the *fimA* virulence alleles by natural competence. Using an *in vitro* competence assay, we were able to generate *fimA* allele replacements in four distinct genetic lineages of *P. gingivalis*, at frequencies between 10^−5^ and 10^−7^. In all recombinants, we detected a complete exchange of the entire *fimA* allele, and exchange of DNA between other genes of the *fimA* genetic locus was also identified. In order to test the global impact of *fimA* genotypic variations on pathogenicity, we exchanged three of the main genotypes of *fimA,* types I, III,and IV, into an isogenic background, *P. gingivalis* strain ATCC 33277 (33277), and quantified major virulence attributes associated with fimbriae. We found that each allele type imparted a distinct set of behaviors to the parent strain; most strikingly, the substitution of the type IV allele from strain W83 into strain 33277 converted the 33277-model strain into one phenotypically more similar to the W83 model strain. These studies reveal genetic strategies potentially utilized by pathogenic flora to modify their interactions with the host, allowing them to ultimately tip the homeostatic balance to their favor in the challenging host environment.

## Results

### The *fimA* Gene Region can be Transferred between *P. gingivalis* Strains

**Figure 1:**
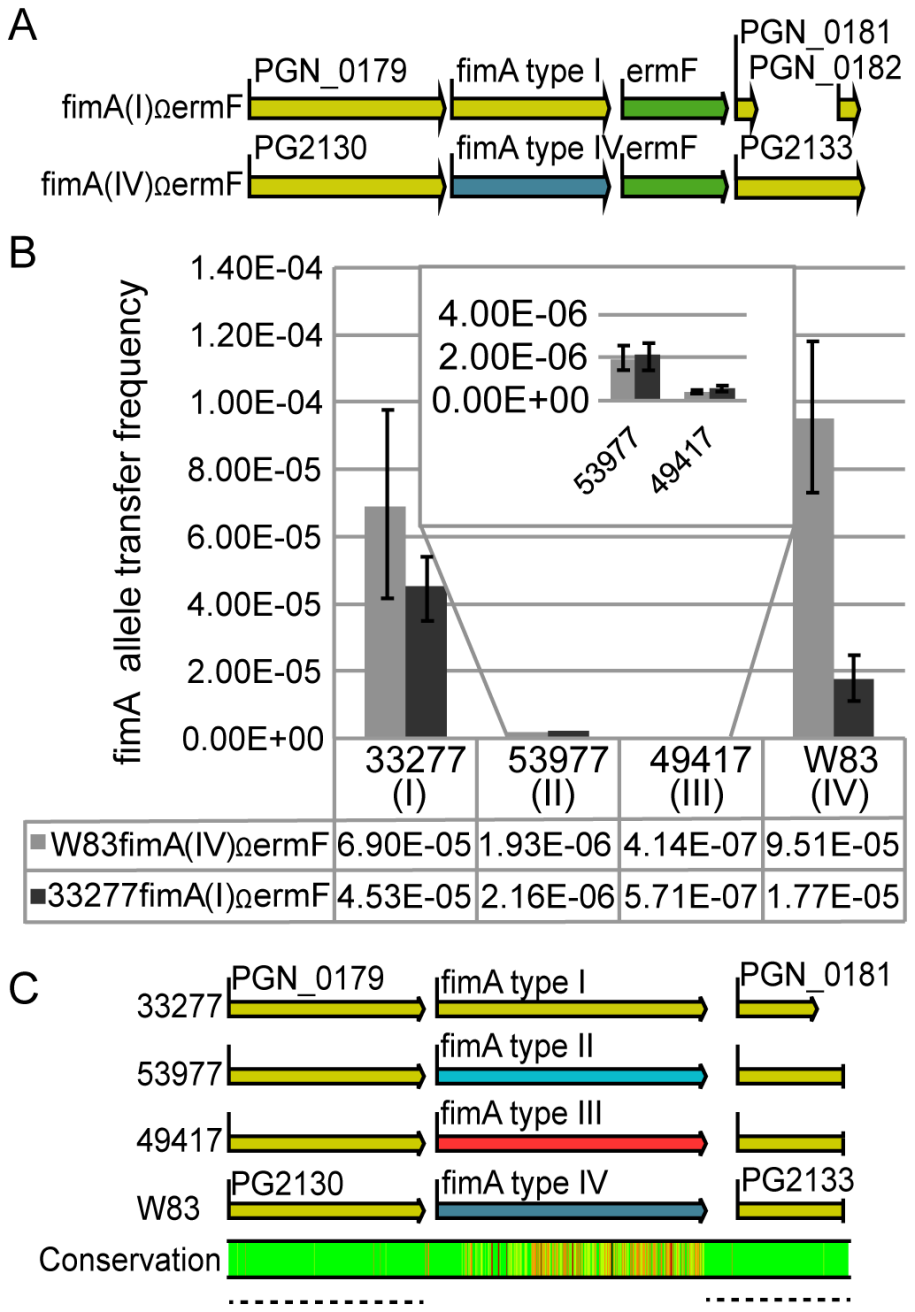
*fimA* allele types are actively exchanged via natural competence. (A.) Genomic constructs for the donor strains 33277 (top) and W83 (bottom). Donor strains contained the genomic insertion *ermF* (green) directly downstream of *fimA* type I (yellow) or IV (blue). Open reading frame sizes are proportional; created using CLC Genomic Workbench 6, from sequence accession numbers NC_010729 (33277) and NC_002950 (W83). (B.) The DNA uptake efficiencies of experiments with four live recipient *P. gingivalis* strains, using either the 33277 or W83 dead donor strain. *fimA* allele transfer frequencies are calculated as the number of recovered recombinants (Rif^r^, Erm^r^) divided by the number of input recipient cells. All data are presented as the mean +/− standard deviation (SD) from a minimum of three independent trials performed in triplicate. All recipient strains were rifampin resistant. Statistical comparisons between all groups were done by one-way Anova. (C.) DNA sequence alignment of the *fimA* recipient alleles from figure 1B. Degree of conservation between the four sequences is shown, with a scale from green to red representing 100% to 0% conservation. Regions responsible for genetic recombination are underlined by dashed lines, and are >98% identical between any donor and recipient DNA sequence. DNA sequences from strain 53977 and 49417 are available at GenBank accession numbers KF770042 and KF770043.

In previous studies, our research group demonstrated that chromosomal DNA is readily exchanged between different strains of *P. gingivalis*, using active uptake of free eDNA, and to a lesser extent by conjugation [44], [46]. This work showed that divergent strains *P. gingivalis* 33277, ATCC 53977 (53977), and W83 were capable of assimilating the genomic DNA marker *PG1244*Ω*tetQ* with no significant bias against DNA donated by other strains [44], [46]. In these previous studies, the DNA containing our transfer marker was homologous between strains of *P. gingivalis*, and the *tetQ* marker was inserted downstream of *PG1244*, in an intergenic region (W83 NC_002950). Having shown that transfer of “biologically neutral” DNA is possible in *P. gingivalis*, in this study we investigate if the *fimA* allele types can be exchanged by natural transformation into different strain backgrounds. In order to test this, we created 33277 and W83 donor strains with chromosomal *fimA(I)*Ω*ermF* or *fimA(IV)*Ω*ermF* as the transfer marker, respectively (Figure 1A). These construct loci are genetically identical for 1 kb upstream and downstream of *fimA* in strains W83 and 33277, except for a single base nonsense substitution in PGN_0181 (*fimB* [50], [51]). A DNA transfer assay with dead donor cells was used to determine if strains 33277 (*fimA* type I), 53977(*fimA* type II), 49417(*fimA* type III), and W83 (*fimA* type IV) are competent for fimbrial allele exchange and to compare the resulting transformation frequencies.

All four *P. gingivalis* strains produced recombinant *fimA* transformants from both donor types (Figure 1B). Strains 33277 and W83 were transformed at significantly higher rates (10^−5^) than either 54977 (10^−6^) or 49417 (10^−7^). Interestingly, while strains 53977 and 33277 were previously found to have similar transfer frequencies for *PG1244*Ω*tetQ* [44], transfer efficiency of *fimA* into strain 53977 is reduced 21-fold for *fimA*(I) and 36-fold for *fimA*(IV) relative to 33277. Strain W83 showed a significant preference (P<0.0001) for recombining with its own *fimA*(IV) versus the *fimA*(I) allele (Figure 1B, column 4), and 33277 also showed a slight, but not significant (P = 0.19), preference for *fimA*(IV). Strains 53977 and 49417 had significantly lower transformation frequencies relative to strains 33277 and W83, but there were no differences in transformation frequency between allele type I and type IV.

Although complete genome sequences for strains 33277 and W83 are available, only a limited amount of genomic sequence is available for strains 53977 and 49417. To ensure that differences in homology upstream and downstream of the *fimA* alleles in strains 53977 and 49417 did not account for the differences in transformation efficiencies, we submitted flanking DNA from these strains for DNA sequencing, allowing us to compare DNA sequence conservation 872 bp upstream and 634 bp downstream of the *fimA* gene (Figure 1C; GenBank accession numbers KF770042 and KF770043). In the upstream 872 base regions, the largest difference was between strain 33277 and W83, which are 98.5% identical with no gaps in the sequence. This represents a 13 bp difference between DNA regions, and the differences were found as single point mutation differences, and not clustered together. The most similar regions were between 33277 and 53977, which were 99.3 percent identical, with no gaps and only 6 base pairs different. Comparison of the downstream flanking regions found that all sequences were greater than 99% identical to each other. Thus, significant sequence homology is found flanking the *fimA* gene, and it seems unlikely that homologous recombination underlies the differing transformation frequencies.

**Figure 2:**
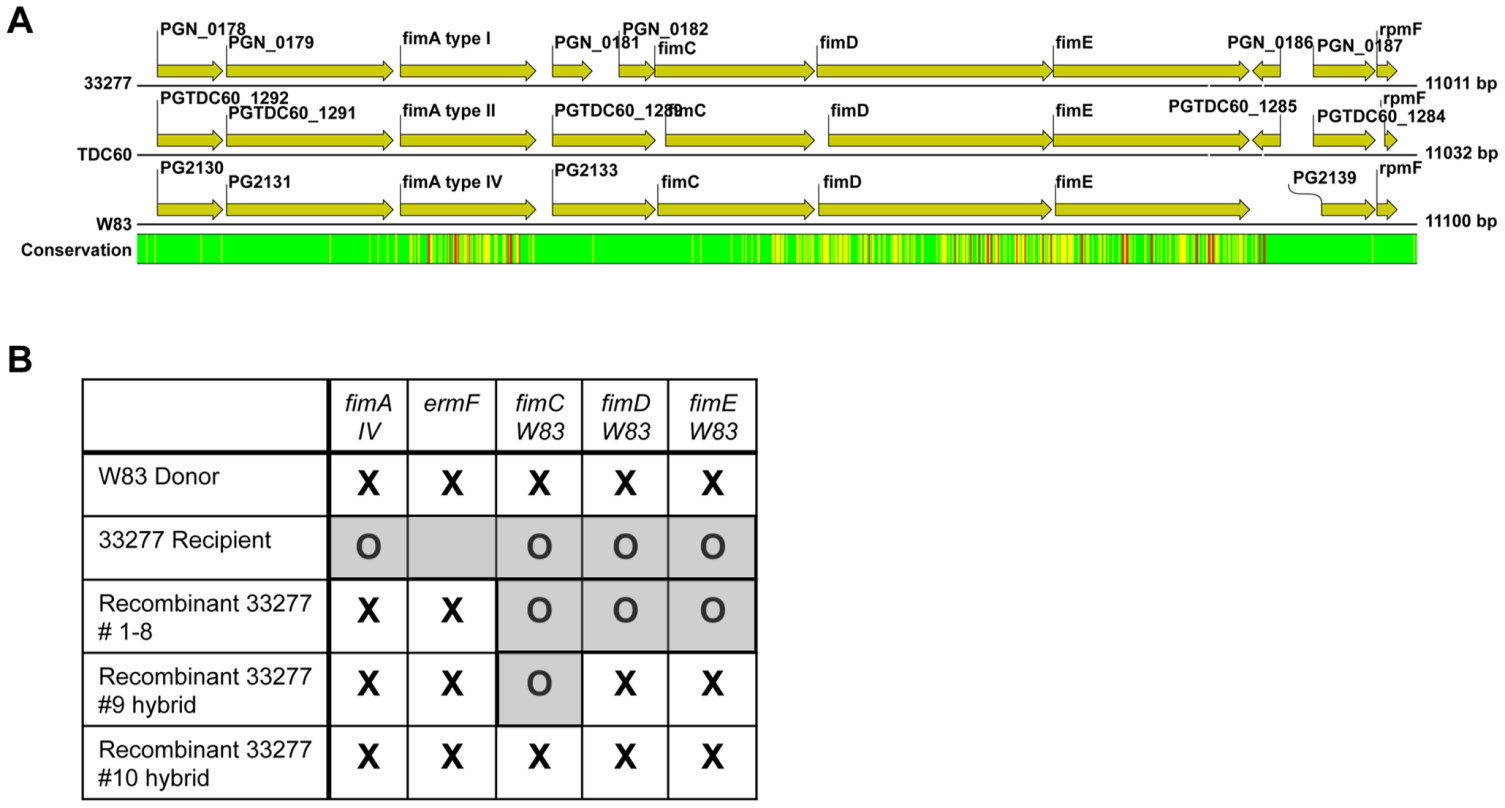
*fim* region able to swap during natural competence despite limited conservation regions. (A.) Complete *fim* gene region (*fimA-fimE*) and flanking genes are shown for sequenced *P. gingivalis* strains 33277*fimA (I*), TDC60 *fimA (II*), and W83 *fimA (IV*). Overall sequence conservation between all strains is displayed. Green indicates the most conservation while yellow, orange, and finally red signifies the least DNA conservation between strains. Distances are shown in base pairs. Figure modified from CLC Genomic Workbench 6, accession numbers 33277(NC_010729), TD60 (NC_015571), and W83 (NC_002950). (B.) Chromosomal preparations from a random sample of ten recovered recombinants (#1–10) from the dead donor assay (donor W83, recipient 33277) were tested for the presence of the donor *fimA* type IV allele using PCR amplification. In addition, to determine if regions further downstream of *fimA* were also swapped, the recombinants were tested for the presence of donor *fimC, fimD*, and *fimE*. ‘**X**’ indicates the presence of the donor gene and ‘**O**’ indicates the presence of the recipient gene in the tested strains.

A random sample of 50 transformants were tested by allele-specific PCR to ensure that *ermF* was not inserted in the absence of the new *fimA* type. In all cases, the entire *fimA* donor allele was present in the transformant, requiring that a minimum of 800 bp of DNA was exchanged within the *fimA* gene. Downstream of the *fimA* allele is an operon encoding *fimC*, *fimD*, and *fimE*. Genetic alignment of the *fim* loci from the three sequenced *P. gingivalis* genomes reveals that, similar to *fimA*, the *fimCDE* region is genetically variable (Figure 2A) [50]–[52]. We selected ten 33277 transformants from a W83 dead donor assay, and used PCR to determine if the *fimCDE* regions were exchanged. Eight transformants contained the *fimA* (IV) allele and *ermF* from the W83 donor, but retained the *fimCDE* region from the 33277 recipient. In the last two transformants, the *fimA* (IV) allele and *ermF* from the donor were present; in addition, recombinant nine had *fimDE*, and recombinant ten had *fimCDE* from the donor (Figure 2B).

Taken together, these results indicate that *fimA* alleles can be exchanged via natural competence but the transformation efficiency between strains varies significantly. This variability does not appear to be dependent on a lack of DNA identity for homologous recombination. In our survey of transformants we detected complete exchange of alleles, thus no hybrid *fimA* genes were produced at a detectable frequency. However, in a preliminary screen we were able to detect allele swaps of *fimCDE*, implying that the entire *fim* locus may be considered “modular” and allele swapping within the region may act to fine-tune fimbrial functions.

### Characterization of FimA Protein Expression in Exchange Mutants

**Figure 3:**
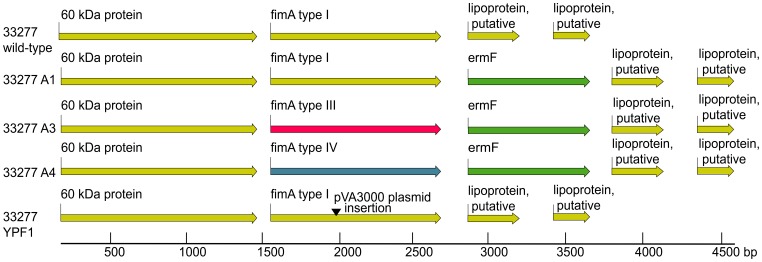
*fimA* allelic replacements in strain 33277. Gene arrangement for parent strain 33277, allelic replacement strains A1, A3, A4, and FimA minus strain YPF1 are shown. Fusion PCR with homologous recombination was used to replace the *fimA* I open reading frame with the same or different *fimA* alleles (indicated by change in color) and to insert the *ermF* marker (green) in the parent 33277. The *fimA* minus mutant (YPF1) shows the insertion location of disruption plasmid pVA3000 (black arrow). Distances are shown in base pairs. Figure generated using CLC Genomic Workbench 6, with DNA sequence from strain 33277 using GenBank accession number (NC_010729).

Our genetic characterization of the natural allele swaps revealed that a complete *fimA* gene exchange occurs. We wished to test the phenotypic changes resulting from this allele replacement, however additional gene exchanges in the *fimCDE* region were detected in some transformants, and other genetic events could have occurred outside the *fim* genetic locus. To test the effect of only *fimA* allelic differences, we used fusion PCR to introduce three different genotypes of *fimA* marked with *erm*F into an isogenic background, strain 33277. The *fimA* open reading frames for types I, III, and IV were PCR-amplified from strains 33277 (I), 49417(III), and W83 (IV), and linked to the *ermF* marker using fusion PCR, and constructs confirmed by sequencing. These constructs were subsequently transformed into strain 33277, creating allelic replacement strains A1, A3, and A4 (Figure 3). These strains retain all upstream and downstream sequence from strain 33277, thus the allele substitutions represent start codon to stop codon DNA only. The A1 construct is a control strain, to account for introduction of the *erm*F marker downstream of *fim*A.

**Figure 4:**
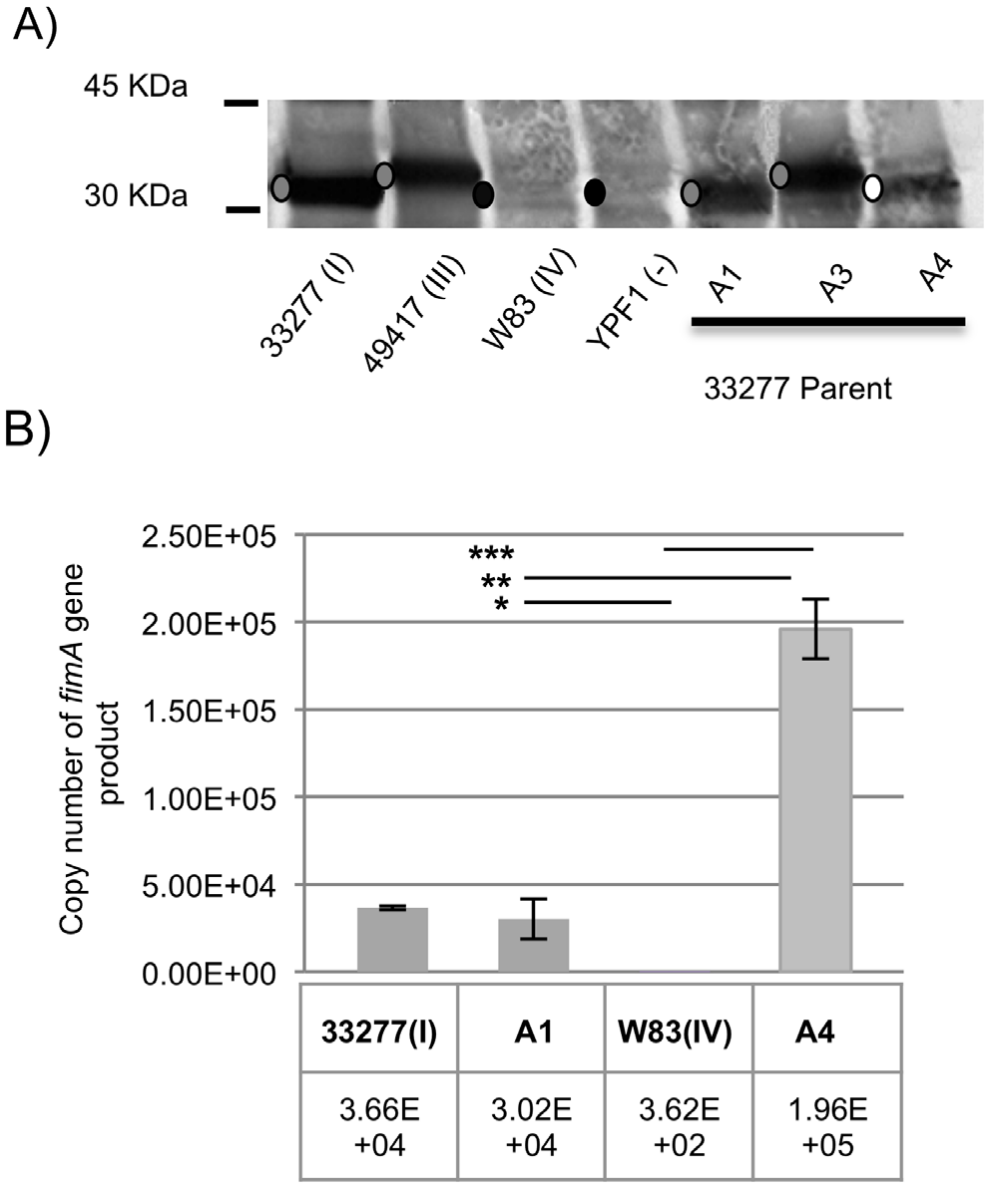
Differential expression of FimA proteins and *fimA* from parent strains and mutant *fimA* swaps. (A.) Outer membrane fractions were subjected to SDS-PAGE, and immunoblots were developed with polyclonal anti-FimA antibodies. Grey dot indicates positive presence of FimA protein. Black dot indicates non-detectable amounts of FimA protein. White dot indicates weak detection of FimA protein within the A4 strain. (B.) Total RNAs were extracted from strains and expression was determined using rtPCR analysis. The transcript level in an individual strain is indicated based on copy number determined from a standard curve of 33277 chromosomal DNA. Strains 33277 (type I), A1∶33277 Δ*fimA* (*fimA*Ω*ermF*, type I), W83 (type IV), A4∶33277 Δ*fimA* (*fimA*Ω*ermF*, type IV). All data are presented as the mean +/− SD from three independent trials performed in triplicate. Statistical difference calculated between A1:W83 *, A1:A4 **, and A4:W83 *** (p<0.001, one way Anova).

Using Western blotting, we compared production of the FimA protein in the isogenic constructs to the allele parents (Figure 4A). The *fimA* allele parents, *P. gingivalis* strains 33277(I) and 49417 (III) produced detectable FimA protein, as assessed with a polyclonal FimA I antibody (Figure 4A). Strains A1 and A3 mimicked their *fimA* allele parent strains 33277 and 49417 with comparable levels of expression and identical migration rates of the protein [27], [53]. Our negative control strain YPF1, a 33277 *fimA* deletion mutant, as expected, was devoid of detectable amounts of FimA. We were unable to detect FimA IV protein from strain W83 (IV), which is not surprising, as previous reports have shown that levels of expression are low in the this strain, and it is antigenically distinct from the type I protein, thus less reactive with FimA I antibody [54], [55]. Interestingly, while expression of FimA IV protein in strain W83 was undetectable, allelic exchange mutant A4 produced detectable amounts of FimA IV protein, implying that much higher levels of this protein are being expressed in the 33277 background than in the W83 background.

Together, these results show that the *fimA* allelic exchange mutants A1and A3 express FimA protein levels comparable with the allelic parent, the A4 strain is expressing more FimA than in the allelic parent, and lastly, the presence of the *ermF* marker in the constructed loci does not appear to hinder expression levels or post-translational processing of FimA protein, as illustrated by comparison of 33277 wild-type to the A1 construct.

### Regulation of *fimA* Allele Expression in *P. gingivalis*

The presence of detectable FimA protein in the A4 strain lead us to investigate whether *fimA* (IV) gene expression was being regulated differently in the 33277 strain background in our model system. RNA preparation and RT-PCR from strains 33277, A1, W83, and A4 were prepared in parallel under the same conditions. A *fimA* gene fragment from all 4 strains was amplified using the same set of primers towards a conserved sequence within the 5′ region. As shown in Figure 4B, *fimA* transcript levels were significantly lower, ∼92 times less expression, in strain W83 when compared to strains 33277 and A1. This is consistent with previous work by Nishikawa *et. al.* [23] showing that the W83 strain lacks a functional *fimSR* two-component system required for activation of *fimA* gene transcription. Intriguingly, the expression level of *fimA*(IV) was highly up regulated relative to *fimA*(I) in the 33277 background, although the promoter sequence is identical in the A1 and A4 strains. This significant increase of almost 6 times the basal level of transcription seen in the parent 33277 and A1 complement strains is striking; one possible explanation is that the FimA IV protein is more potent at binding to *fimA* promoter enhancer sites [24]. This high level of transcription likely explains the detection of FimA IV protein in the A4 strain relative to the parent W83 (Figure 4A).

### Effects of the *fimA* Allele Swaps on Major Fimbriae Surface Expression

**Figure 5:**
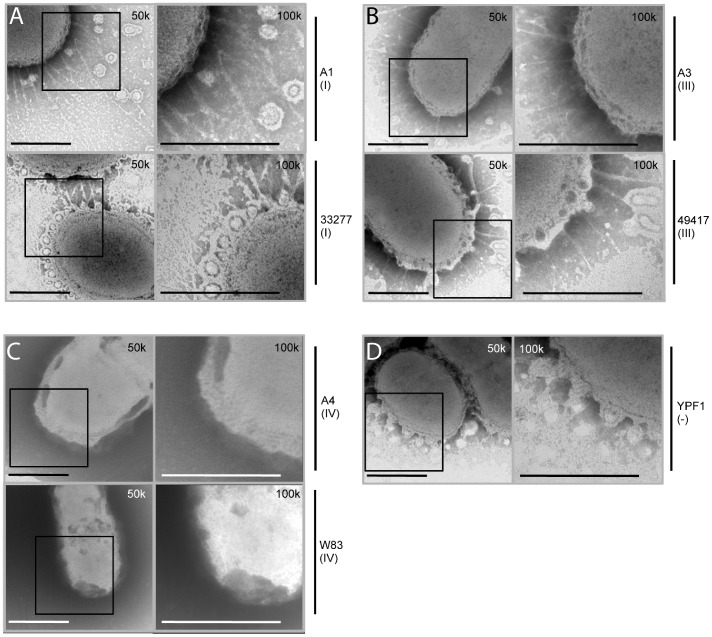
Electron micrographs of *P. gingivalis* surface structures. (A.) *P. gingivalis* strains 33277 A1 and 33277 (both *fimA* type I). (B.) *P. gingivalis* 33277 A3 and 49417 (both *fimA* type III). (C.) *P. gingivalis* 33277 A4 and W83 (both *fimA* type IV). (D.) *P. gingivalis* strain 33277 *fimA* minus, YPF1. Samples were stained with 2% PTA and subjected to transmission electron microscopy, viewed using a JEOL JEM 1010 microscope. Bars = 500 nm. Black box indicates area at 50,000x direct magnification (50 k) that was examined further at 100,000x direct magnification (100 k).

We next examined whether the *fimA* allelic exchange strains showed any observable differences in extracellular fimbria assembly. Surface expression of fimbriae in our strain collection was investigated using transmission electron microscopy (TEM). Strain A1 displayed long, delicate fimbriae and membrane vesicles at the cell surface, similar to that of the parent strain 33277 (Figure 5A). Allele swapped strain A3 also presented with vesicle formation and polymerized fimbriae, which appeared slightly more rope-like at the cell surface, characteristic of the type III FimA fimbriae seen in parent FimA strain 49417 (Fig. 5B). In contrast to strains A1 and A3, allele swapped strain A4 revealed a complete lack of fimbriae at the cell surface, comparable to the exterior seen in strain W83 (Figure 5C). This is consistent with a previous report that found strain W83 to be visibly afimbriate [23]. Nevertheless, protein analysis of the outer membrane fraction from the A4 strain did reveal detectable FimA protein levels (see figure 4A). This is consistent with work by others, showing that type IV monomers are expressed on the cell surface, but polymerization into visible fimbriae does not occur [21]. Also noteworthy was the lack of membrane blebs in A4, again more similar to the *fimA* allele parent than the 33277-background strain. In contrast, our FimA minus mutant YPF1 lacked major fimbria, as expected, but displayed an abundant amount of vesicles at the cell surface (Figure 5D). These data mirror the observed protein levels and furthermore reveal that substituting the FimA subunit alone results in unique characteristics within the extracellular fimbriae display, and the fimbrial type is important for production of another virulence factor, membrane vesicles.

### Aggregation Altered by *fimA* Type III and IV Exchange

**Figure 6:**
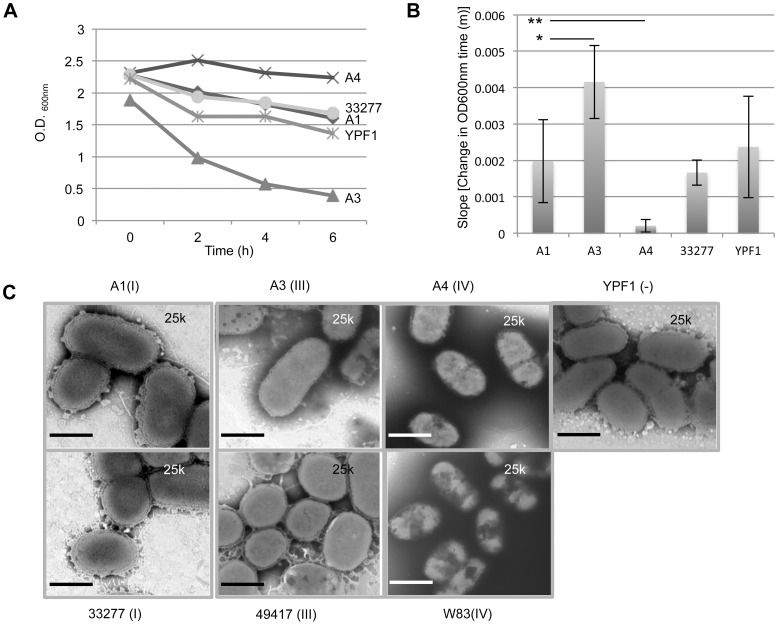
Auto-aggregation and membrane blebbing are altered with introduction of type III or IV *fimA*. (A) A quantitative aggregation assay is shown for the parent 33277, strains A1, A3 and A4, and YPF1. The changes in optical density (O.D.600) were monitored over a period of 6 h. A decrease in O.D. indicates more clumping or aggregation of cells at the bottom of the culture tube. (B) Calculated average slope of auto-aggregation from (A) is shown. All data are presented as the mean average slope +/− SD from five independent trials performed in triplicate. Comparision of all groups by one-way Anova found no significant differences between 33277, A1, and YPF1. Significant statistical differences are shown for A1:A3 *p = 0.01, and A1:A4 **p = 0.003. Presence of type III FimA lead to a significant increase in auto-aggregation, while type IV FimA showed almost complete loss of aggregation. (C) *fimA* allele swap strains (A1, A3,A4) and FimA minus strain YPF1 were observed for cell clumping, membrane blebbing, and vesicle formation using transmission electron microscopy (upper panels). The corresponding *fimA* allele parent strains (33277, type I; 49417, type III; and W83, type IV) are shown below their corresponding swap strain. All strains appear to actively clump or aggregate with the exception of W83 and A4, consistent with the aggregation assays. Moreover, strains W83 and A4 also show a distinct loss of membrane blebbing and vesicle formation. Samples were stained with 2% PTA and observed by transmission electron microscopy using a JEOL JEM 1010 microscope at 25 K. Bars = 500 nm.

Auto-aggregation enhances *P. gingivalis* interactions with biofilms and environmental surfaces and is driven in part by the presence of outer surface appendages such as fimbria [9]; therefore, we investigated if *fimA* exchange would be sufficient to alter the aggregation phenotype. Strain A1 displayed an aggregation rate that was not significantly different from the parent strain 33277 (Figure 6A, B). Strain A3 had a significant increase in auto-aggregation in comparison to all strains tested. In contrast, the *fimA*(IV) swap A4, which presented as afimbriated, showed the least amount of auto aggregation over a six-hour period (Figure 6A, B). Surprisingly, our FimA minus mutant YPF1 displayed no significant difference in auto-aggregation in comparison to the parent or complement strain A1. As previously noted, unlike the A4 strain, YPF1 produces copious membrane vesicles, which might explain its auto-aggregation ability.

To further characterize the aggregation phenotype, we observed our strains using TEM at 25,000x magnification. Cells were monitored qualitatively for the presence of cell-cell associations. Moreover, we also noted the presence or absence of vesicles and extracellular material. Overall, we found that each of the strains showed association tendencies similar to their parent FimA type strain (Figure 6C). FimA types I, III, and YPF1 all commonly displayed cells in close groups or in physical contact, while FimA type IV strains showed less overall cell-cell associations. Currently, there is not a clear connection between outer membrane vesicle production and *P. gingivalis* aggregation; however, we found it intriguing that unlike the other FimA types, FimA type IV strains showed a distinct lack of outer membrane vesicles and extracellular material, and it is the lack of vesicles that correlates with the lack of auto-aggregation in comparison to YPF1. Taken together, these results indicate that fimbrial allele type significantly impacts production of extracellular material in the form of membrane vesicles and visible fimbriae, and which together can increase or decrease rates of aggregation.

### FimA Types III and IV Enhance Gingival Invasion

**Figure 7:**
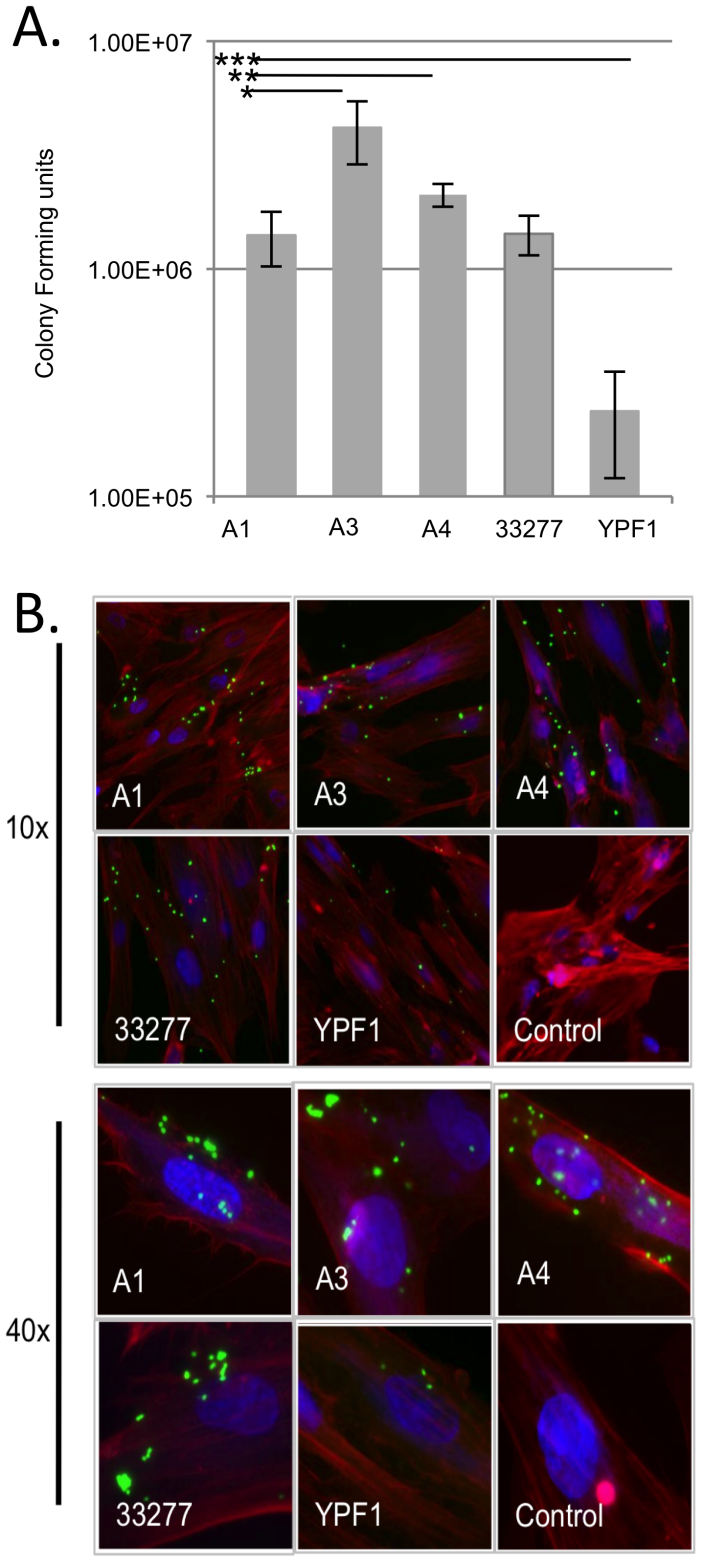
Invasion of human gingival tissue by *fimA* isogenic exchange mutants. A 24-hour invasion of gingival fibroblast cells by *P. gingivalis* was quantitated by an antibiotic protection assay. There is no significant difference in invasion rates between A1 and 33277; YPF1 has a reduced invasion efficiency. Introduction of the type III or type IV *fimA* alleles into 33277 leads to enhanced invasion capability compared to the A1 strain. All data are presented as the mean +/− SD from five independent trials performed in triplicate. Statistically significant differences calculated between A1:A3 *, A1:A4 **, and A1:YPF1 *** (p<0.001; one-way Anova). (B.) Fluorescein (green) labeled *P. gingivalis* cells were added to a monolayer of gingival fibroblast cells (GFC) and incubated for 24 hours. Following incubation, GFC were washed to remove extracellular bacteria. Cells were then fixed and stained with rhodamine phalloidin (red) and DAPI (blue). More bacterial cells were observed associated with GFCs in the A1, A3, and A4 swaps versus FimA minus (YPF1) strains.

Finally, we investigated the impact of *fimA* allelic exchange on bacterial invasion, as internalization and persistence within host cells is dependent on fimbrial type and regulation of *fimA* expression. Fimbrial adhesion to β-integrin and activation of host membrane invagination is critical for internalization. Furthermore, once in the host cell, down regulation and “clearance” of the hair-like surface structures appears to be important for efficient long-term persistence [28]; mutants lacking fimbriae are less efficient at host cell invasion, but once internalized have improved survival rates over time [29]. In order to best replicate the oral niche host environment, we used low-passage, primary gingival fibroblast cells (GFC) for our invasion studies, and invasion efficiency was measured at twenty-four hours, to represent the cumulative effect of internalization and survival. Invasion efficiency was monitored by comparing recovered colony forming units (CFUs) following an antibiotic protection assay (Figure 7A) and by observation of bacteria and host cells via fluorescence microscopy (Figure 7B). The A1 control strain showed invasion levels indistinguishable from the parent 33277. Substitution of *fimA* type I with type III (A3) or type IV (A4) significantly increased invasion of GFCs; whereas FimA minus strain YPF1 showed significantly less invasion than all strains tested. Based on these results, we see that production of FimA type III results in higher levels of invasion; and while strain A4 has a significantly different phenotype from A3 in fimbrial production, this strain is also able to produce a high-level of cell invasion and survival.

### FimA Protein Motif Analysis

**Figure 8:**
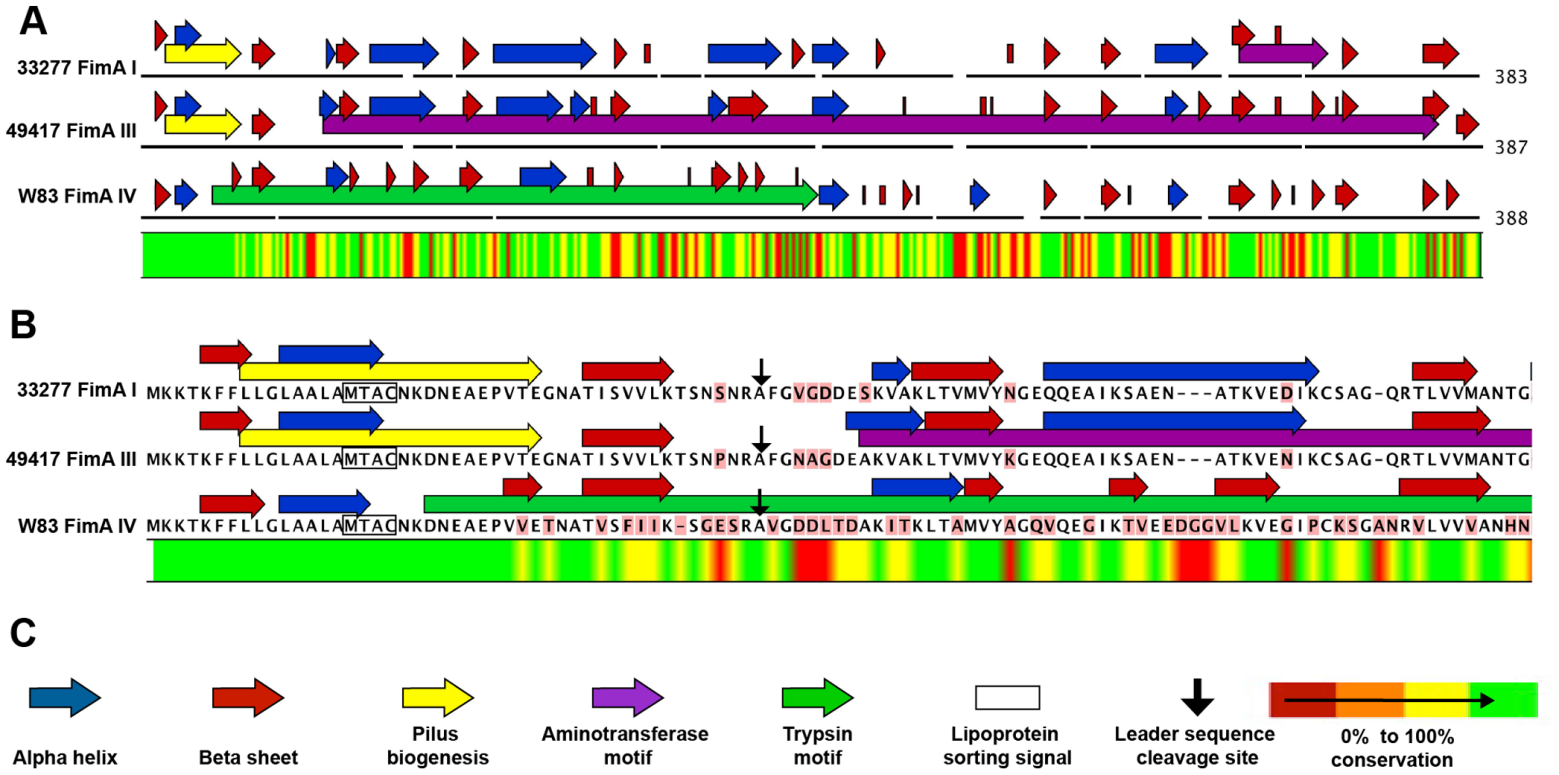
Bioinformatics analysis of *P. gingivalis* FimA protein alleles. (A.) Predicted proteins motifs are shown for FimA type I, III and IV, from *P. gingivalis* strains 33277 (I), 49417 (III), and W83 (IV), respectively. Protein motif analysis and alignment were performed with CLC Genomics Workbench software version 6. (B.) Alignment of the N-terminal regions of fimbrial proteins. Boxed sequences (MTAC) are the lipoprotein sorting signals. Black arrows indicate the predicted gingipain cleavage sites of the leader peptide. Residue conservation between all strains is displayed as a color bar below the alignment. Red highlighting of individual residues shows deviation from the aligned consensus. (C.) Guide to color and symbols in A and B. The pilus biogenesis symbol represents a match to the pfam Pilus biogenesis CpaD protein (pilus_cpaD) Accession: PF09476. The aminotransferase represents a match to pfam Aminotransferase class I and II, Accession: PF00155. The trypsin symbol represents a match to pfam Trypsin, Accession: PF00089. Sequence conservation between all strains is displayed below the alignment. Green indicates the most conservation while red signifies the least conservation between strains.

In order to investigate the protein expression and processing differences between fimbriae, we compiled an alignment and protein motif analysis of the four FimA types, using the “create alignment “and “pfam domain search” of CLC Genomics Workbench version 6, respectively (Figure 8). All four fimbria types are identical over the first 26 N-terminal amino acid sequences. FimA types I and III have a potential pilus biogenesis motif located near the N-terminus (Figure 8A, yellow arrows). All FimA types have conserved amino acid sequences responsible for recognition and cleavage of the leader peptide (Figure 8B), and cleavage of the leader sequence at residues R-46 and A-47 is consistent with the type I FimA size observed on our Western blots (36.5 kDa, Figure 3A). Interestingly, although FimA I and III have the same predicted molecular weight before and after leader processing, there are some apparent differences in protein modification between the strain types. Previous studies have shown that strain 49417 removes the leader peptide, but the fimbrial monomer of type III naturally migrates more slowly on SDS-PAGE gels in comparison to the mature type I protein [56], [57].

The FimA IV protein is the least conserved of the group, and its amino acid sequence diverges from the other two types beginning at residue 26 (Figure 8B), however the leader peptide cleavage site is located at the conserved residues R-45 and A-46. FimA IV lacked an identical match to the motif for pilus biogenesis, but was uniquely predicted to have a trypsin-like enzyme motif (Figure 8AC) that spans approximately half of the protein starting near the N-terminus. Further investigation of the motif identified catalytic residues consistent with trypsin-like peptidases (data not shown) [58]. Hypothetically, the presence of peptidase activity within the monomer could be a mechanism to cleave assembled units and limit fimbrial polymerization. Collectively, we demonstrate that each *fimA* genotype expresses a distinct protein structure, and that this variability at the protein level likely contributes to our observations on phenotypic variability between strains.

## Discussion

Together these results demonstrate that genetic re-assortment of virulence-alleles provides an active means for bacteria to adapt and persist in the host environment; and that even minor changes in genetic content can result in significant alterations in phenotypic behaviors. These behaviors may be directly related to pathogenic interactions or alternatively present changes consistent with a more commensal relationship with the host environment. Bacteria that maintain a flexible genome are thought to have a distinct survival advantage in challenging and rapidly changing environments. One possible explanation for this adaptability is the hypothesis that bacteria can access the entire pan-genome of their species, dramatically increasing their genetic repertoire (Reviewed in [59], [60]). This variability might provide modification and/or additional pathways and functions that are not essential for bacterial growth but overall confer a selective advantage in a particular environment. We suggest that strains with a greater ability to swap major virulence factor allele types from their pan-genome may have a clear fitness advantage, access to a larger genetic repertoire, and potentially the best overall ability to persist in the host environment. Based on our previous identification of natural competence in multiple strains of *P. gingivalis*, and on the presence of multiple *fimA* alleles within the *P. gingivalis* pan-genome, we predicted that individual strains could exchange their FimA type with neighboring strains to generate a diverse pool of phenotypic behaviors, and thus modify their interactions with the host environment. Our results demonstrate that *fimA* gene expression, post-translational processing, fimbrial elaboration, auto-aggregation, invasion, and vesicle formation were all significantly impacted as the result of changes in the *fimA* allele type.

Four distinct strains were shown to be capable of exchanging *fimA* allele types using natural competence. Yet, there was a clear significant difference among the rates of transformation frequency between each of the strains tested. We showed previously that strains 53977 and 33277 swap a “biologically neutral” gene-marker with nearly identical frequency [44]. Yet, when presented with the option to exchange *fimA* types, there was a significant decrease in strain 53977’s ability to accept a new *fimA* type relative to strain 33277. Strain 53977 possesses a *fimA* type II allele; it is possible that this strain has less tolerance for recombination of non-homologous sequences than 33277. However, 53977 showed no difference in transformation frequencies between a donor *fimA* type I and a donor *fimA* type IV. An alternative interpretation is “biological compatibility” with the host strain metabolic system. Introduction of a new fimbrial subunit might be a stressful event in some strains. This could indirectly result in decreased transformation frequencies.

The majority of studies to date that found differences between strains with distinct fimbrial types did not account for the variation in the genetic backgrounds of the strains as a possible influence. More recent publications have begun to introduce fimbrial genes into control strains to provide better understanding of the features of different *fimA* types. In a relatively recent study Kato et. al. exchanged the fimbrial region from 33277 (type I) and OMZ314 (type II) including *fimA*, *fimB, fimC*, and *fimD* plus several upstream genes [61]. Their fimbrial locus swaps showed that the type II fimbrial region improved 33277 adhesion and invasion of epithelial cell lines. In our study, we found that substitution of *fimA* type III or IV into 33277 resulted in increased invasion of gingival cells, however our study paired FimA III or IV with FimBCDE from 33277. Deletion of *fimC* or *fimD* has been shown to impact auto-aggregation and binding ability [19], thus the ability of the adhesins FimC and FimD to associate with a specific FimA type could be anticipated to significantly impact these behaviors. We were able to detect exchange of *fimC*, *fimD* and *fimE* alleles between 33277 and W83, thus the modular re-assortment of this locus is biologically possible.

Assessment of the FimA exchange strains by Western blot and TEM revealed that strains A1 and A3 displayed monomer expression and extracellular fimbriae polymerization. Strain A4, however, did not display any observable extracellular fimbriae, in spite of significant up regulation of transcription. The low production of FimA(IV) in strain W83 was previously shown to be due to a non-functional FimS histidine kinase, and consistent with our work, transfer of the *fimA(IV)* allele into 33277 in that study also provided detectable levels of monomer subunit, but not fimbrial polymerization at the surface [21]. In contrast, Nagano et. al. introduced a plasmid-borne *fimA(IV)* allele into a 33277 strain constructed to lack the entire *fim* locus, including *fimBCDE* [62]. In that context, they were able to detect levels of FimA IV monomer equivalent to isogenic strains with other FimA types, and the FimA IV monomer polymerized into visible surface fimbriae. The FimB protein is not essential for assembly of the FimA monomers into the polymeric fimbriae, but when present it does appear to regulate the length of the final appendage [20]. Strain 33277 encodes a truncated *fimB* gene; it may be that for the FimA IV monomer the presence of FimB or even a partial FimB protein destabilizes its ability to polymerize into a visible structure. Bioinformatics analysis revealed an additional unique feature of FimA IV, a putative trypsin-like motif. Protein auto-lysis could contribute to reduced levels of FimA IV protein and/or the presence of modified FimA IV subunit could also decrease fimbriae polymerization at the cell surface. Therefore, the production of polymerized fimbriae is regulated at several points and possession of the *fimA* IV allele can result in significant differences in surface appearance and function.

Functional analysis of the FimA exchange strains revealed that auto-aggregation was changed in response to swapping the *fimA* allele type. Strain A1 had similar aggregation rates compared to the parent 33277, while strain A3 showed significantly enhanced aggregation, and strain A4 with no polymerized major fimbriae lost aggregation ability. Surprisingly, the FimA minus mutant displayed auto-aggregation rates similar to that of the A1 control strain and parent 33277. The presence of prolific membrane blebbing in YPF1 appears to compensate for the lack of fimbriae and allow for normal levels of auto-aggregation, but blebbing did not compensate for lack of fimbriae during invasion of host cells. It is curious that neither the A4 strain nor the YPF1 mutant produce intact fimbriae, but only YPF1 produces membrane blebs. In Nagano et. al. the authors mention that the 33277 mutant strain lacking the entire *fim* locus has an “unstable” outer membrane with excessive vesicle formation [20]. Vesicle formation is an important factor in the pathogenesis of a number of gram negative bacteria (Reviewed in [63]). Earlier work by Zhou et al. [64] has looked at the composition of the vesicles produced from various *P. gingivalis* strains and they predicted that the contents of these vesicles may be important in provoking the host inflammatory response. Loss of major membrane blebbing and vesicle production such as exhibited by W83 and the A4 strain may be another strain-specific feature that regulates the localized immune response allowing for further colonization of host tissue unnoticed.

Invasion is a complex, multistep process, with orchestrated temporal expression of several virulence-associated genes [4], [65], [66]. Invasion was increased relative to 33277 in the A3 and A4 strains. This is particularly noteworthy because in all other assessed characteristics the A3 and A4 strains are significantly different. It may be that A3 is more efficient at early steps such as adherence, but due to lack of fimbriae, A4 may survive better once internalized. This highlights that adherence, internalization, survival, and replication each take place during the ‘invasion’ process and highly efficient abilities in one stage may compensate for decreased abilities elsewhere.

Extensive clinical studies have found an overall correlation between fimbrial type and the presence of gingivitis and periodontitis, namely *fimA II* and *fimA IV* genes [34], [37], [38], [67]–[70]. Nevertheless, genetic surveys have continued to find the other FimA allele types, type I, Ib, III, and V persisting in the *P. gingivalis* population [30], [71]. This brings about the question: why maintain these other FimA types? The pan-genome presents the idea that species maintain both a ‘core’ genome and a flexible, ‘dispensable’ genome, and sampling DNA from the flexible genome drives adaption to the changing environment (reviewed in [59], [60]). We demonstrate here that even limited genetic changes in a single gene resulted in widespread biological changes, impacting behaviors directly related to survival in the host. It still remains unclear what cues or signals are responsible for possibly triggering these virulence allele exchange events. An increase in host cellular factors, nutrient levels, and/or bacterial cell density could prompt genetic exchange events leading to a short-term energy cost but long-term fitness advantage by generating strains with more appropriate allele combinations. Taken together, this work begins to provide fundamental insight into some of the key molecular mechanisms bacteria may use to access their pan-genome, leading to enhanced survival and adaptive virulence.

## Materials and Methods

### Ethics Statement

Human gingival tissue explants were obtained with signed consent from donors, and used for isolation of primary gingival fibroblasts. The protocol was reviewed and approved by the Committee for the Protection of Human Subjects (CPHS) of the University of Texas Health Science Center at Houston (IRB: HSC-DB-10-0575).

### Bacterial Strains and Growth Conditions

*Porphyromonas gingivalis* strains were grown anaerobically at 37°C in a Coy anaerobic chamber in Trypticase Soy Broth (TSB) culture media supplemented with hemin and menadione, as previously described [44]. TSB blood agar plates (BAP) were made with the addition of 5% sheep’s blood and1.5% agar. Selection for antibiotic resistant *P. gingivalis* was with 10 µg/ml erythromycin and 30 µg/ml rifampin. For strains used in this study please refer to Table 1 [44], [72].

**Table 1:** Bacterial strains used in this study.

Strain	Antibiotic Selection	Mutation	Strainabbreviation	Source
*P. gingivalis* W83			W83	Ann Progulske-Fox lab, University of Florida, 2003
*P. gingivalis* ATCC 33277			33277	Richard Lamont lab, University of Florida, 2003
*P. gingivalis* ATCC 53977			53977	Richard Lamont lab, University of Florida, 2003
*P. gingivalis* ATCC 49417			49417	Richard Lamont lab, University of Florida, 2003
*P. gingivalis* W83	Rif^r^		W83 Rifr	[44]
*P. gingivalis* ATCC 33277	Rif^r^		ATCC 33277 Rifr	[44]
*P. gingivalis* 53977	Rif^r^		53977 Rifr	[44]
*P. gingivalis* 49417	Rif^r^		49417 Rifr	[44]
*P. gingivalis* ATCC 33277 - A1	Rif^r^	FimA exchange Δ*fimA1*(Ω*fimA(I)::ermF*)	A1	This study
*P. gingivalis* ATCC 33277– A3	Erm^r^	FimA exchange Δ*fimA1*(Ω*fimA(III)::ermF*)	A3	This study
*P. gingivalis* ATCC 33277– A4	Erm^r^	FimA exchange Δ*fimA1*(Ω*fimA(IV)::ermF*)	A4	This study
*P. gingivalis* ATCC 33277–YPF1	Erm^r^	FimA deficient (disruption) pVA3000 *fimA(I)::ermF*	YPF1	[72]
*P. gingivalis* W83	Erm^r^	FimA *fimA(IV)*Ω*ermF*	W83 FimA Ermr	This study

### Molecular Biology and Bioinformatics

Total DNA was purified from *P. gingivalis* using the Promega Wizard genomic DNA purification kit (Promega, Madison, WI). DNA amplification by PCR was performed using the high-fidelity ExTaq polymerase (TaKaRa Bio, Inc., Otsu, Shiga, Japan). Primers were synthesized by Sigma-Aldrich, Inc. (Sigma Life Sciences, The Woodlands, TX) or Integrated DNA Technologies (IDT); Coralville, IA. Primers used in this study are listed in Table 2 in the supplemental material. DNA sequencing was performed by SeqWright DNA Technology Services (Houston, TX). Sequence assembly and DNA and protein analysis were done with CLC Genomics Workbench, v6 (CLC bio, Cambridge, MA). The *fimA* upstream and downstream DNA sequences from strains 53977 and 49417 were submitted to GenBank under accession numbers KF770042 and KF770043, respectively.

**Table 2:** PCR Primers used in this study.

Primer Name	5′ to 3′ direction
FimA fusion A	TGGGACTTGCTGCTCTTGCTATGA
FimA fusion B	CTCTCACTCTGAATGGATAAAGTTTGCTAGTCGTTTGACGGGTCGATTACCA
FimA fusion C	AAGTCGGGTGGTTGTCAAGATGATTTTTCTCCCTGTATTCATTCTCT
FimA fusion D	TCCCCGTTTTGTATCTGT
ErmF Fwd	AGCAAACTTTATCCATTCAGAGTGAGAG
ErmF Rev	AAAAATCATCTTGACAACCACCCGACTT
FimA fusion Fwd	AAGACAACGAGGCAGAA
FimA fusion Rev	GGTATAAGTGTCGGGAG
FimA Fwd rt-pcr Type I-IV	AGTTTTTCTTGTTGGGACTTGC
FimA Rev rt-pcr Type I-IV	GGTTCTGCCTCGTTGTCTTT
FimA Fwd Type I	CAGCAGGAAGCCATCAAAT
FimA Rev Type I	CTTAGGCGTATAATTGCTGTC
FimA Fwd Type IV	ACGGCGGAGTCCTTAAAG
FimA Rev Type IV	TTTGTCCTTGGGTAATAGC
FimC Fwd Type 33277	TCCTTGTACCCTATGTGGC
FimC Rev Type 33277	TGGGGGTTGATATTCTTGTCA
FimC Fwd Type W83	ACTTGTTTCCATTGTTCCC
FimC Rev Type W83	TGTCCTGCCCCGGTATTG
FimD Fwd Type 33277	GTGCGTGAAGACATAGAGTC
FimD Rev Type 33277	CTCGCATTCGGCTCAAAA
FimD Fwd Type W83	GAGGAGCTCTCTATTTGCG
FimD Rev Type W83	GGATAGAATTGGCAGGTG
FimE Fwd Type 33277	TACAGGCTCTTTTCCTCAG
FimE Rev Type 33277	GGCCGAAATTTGAGTGGTT
FimE Fwd Type W83	TTCAAACCTGTCGGTCCT
FimE Rev Type W83	CCTGCGTCTTATGTACCT

### *fimA* Allelic Replacement in Strain 33277

Allelic exchange mutants of *P. gingivalis* ATCC 33277 were generated by creating fusion PCR products according to a protocol derived from the work of Kuwayama et al. [73]. Briefly, the *fimA* open reading frame from bacterial strains 33277(*fimA* type I), 49417 (*fimA* type III), or W83 (*fimA* type IV) was amplified using PCR primers *fimA fusion A* and *fimA fusion B*, which are designed to anneal in conserved regions at the start and stop codon of *fimA* (Table 2). Then, using template DNA from strain 33277 only, an 800 bp region immediately downstream from *fimA* was also amplified, with primers *fimA fusion C* and *fimA fusion D*. Primers *fimA fusion B* and *fimA fusion C* contained 30 bp reverse complement sequences for a *P. gingivalis ermF* selectable marker. To create a final fusion product, equal ratios of purified PCR fragments for a *fimA* allele, the *ermF* marker, and the 800 bp downstream region were mixed and used in a final PCR reaction with nested primers *fimA fusion Fwd* and *fimA fusion Rev*. Three separate PCR fusion reactions were done, creating constructs with each of the *fimA* types. Full fusion PCR products were sequenced and electroporated directly into ATCC 33277 without cloning. Transformants were selected on erythromycin and confirmed by allele-specific PCR, and amplification and sequencing to confirm the integrated construct. The allelic replacement strains in the 33277 background are subsequently referred to as A1 (type I), A3 (type III), and A4 (type IV). Primers used in this study are listed in Table 2.

### Transmission Electron Microscopy

For transmission electron microscopy (TEM), *P. gingivalis* strains were grown in TSB broth to an OD_600_ of 0.8. Strains were then streaked onto BAP and grown for 2 days anaerobically at 37°C. One hundred microliters of 50∶50 TSB broth:1×PBS (phosphate buffered saline) was added to the isolated colony area of each plate and allowed to sit for 15 min at room temperature. Poly-L-lysine coated, 100-mesh gold formvar carbon grids (Electron Microscope Sciences, Hatfield, PA) were gently placed on top of the moistened colonies and allowed to sit for 5 min. The grids were gently lifted from the plate, stained for 1 min with 2% phosphotungstic acid (PTA, pH 7.0, Electron Microscope Sciences, Hatfield, PA), and allowed to air dry at room temperature. Grids were examined in a JEM 1010 transmission electron microscope (JEOL, USA, Inc., Peabody, MA) at an accelerating voltage of 80 kV. Digital images were obtained using AMT Imaging System (Advanced Microscopy Techniques, Danvers, MA).

### Outer Membrane Associated Protein Isolation

Briefly, bacteria were grown planktonically in TSB to an OD_600_ of 0.8. The bacteria cells were harvested by centrifugation at 8,000×*g* for 10 min. The supernatant was removed and the cell pellets were resuspended in 100 µl of outer membrane extraction buffer [20 mM Tris (pH 7.5), 1% TritonX-100, 20 mM MgCl_2_, 2 mM *N*_α_-Tosyl-L-lysine chloromethyl ketone hydrochloride (Sigma-Aldrich, St. Louis, MO), 1X protease inhibitor cocktail for bacterial cell extracts (AEBSF, 11 mM, Aprotinin,1 mM, Bestatin, 65 µM, EDTA, 50 mM, E-64, 0.15 mM, Pepstatin A, 0.15 mM; Sigma-Aldrich Co.)]. After 30 min incubation at room temperature, cell debris was pelleted by centrifugation at 8,000×g for 10 min. The resulting supernatant was mixed with in 20 µl of 5× Laemmli buffer. Samples were boiled for 5 min and centrifuged at 400×*g* for 5 min, and the proteins were analyzed using SDS-PAGE as described below.

### Protein Analysis and Immunoblotting

Outer membrane isolated proteins were resolved by sodium dodecyl sulfate-polyacrylamide gel electrophoresis (SDS-PAGE) using the Mini-Protean TGX precast 4–15% gel system (BIO-RAD, Hercules, CA). The proteins were transferred to polyvinylidene difluoride membranes (Immobilon P; Millipore, Billerica, MA). The membranes were incubated with rabbit anti-33277 FimA primary antibody in a 1∶1000 dilution 10% dry skim milk in Tris-buffered saline containing 0.1% Tween 20 (TTBS) rocking over night at 4°C. Membranes were washed three times with TTBS and then incubated with a florescent anti-rabbit secondary antibody Li-Core (Li-Core Biosciences, Lincoln, NE) Chemi-IR conjugate IRDye 800CN at 1∶5000 dilution in 1x Odyssey blocking buffer (Li-Core Biosciences). Membranes were washed three times with 1x Odyssey blocking buffer and fluorescence was visualized using the Odyssey CLx system (Li-Core Biosciences). Pre-stained SDS-PAGE Precision Blue Plus Protein Standards ladder were from BIO-RAD.

### *P. gingivalis* DNA Transfer Assays with Dead Donor Cells

*P. gingivalis*-to-*P. gingivalis fimA* DNA transfer assays with a dead donor were performed as previously described [44]. Briefly, *fimA*Ω*ermF* dead donors were created by incubation of bacteria at 85°C for 10 minutes, then rapid cooling on ice. For each experiment, aliquots of dead donors were plated on BAP to ensure 100% cell death. Dead donor cells were mixed with rifampin resistant recipient strains. Recombinant *P. gingivalis* offspring were selected by resuspending the bacterial pellet into TSB and incubating the recovered bacteria for 10 to 14 days anaerobically on BAP containing both rifampin and erythromycin. DNA transfer efficiencies were calculated by dividing the total number of offspring by the number of input recipient bacterial cells. Chromosomal preparations were isolated from recombinants to confirm the presence of the donor *fim* allele types using PCR amplification. Chromosomal DNA was isolated using the Promega Wizard genomic DNA kit. PCR amplification was performed with ExTaq polymerase and the indicated primers (Table 2) using the following PCR program: 94°C 30 sec, 30 cycles: 94°C 10 sec, 55°C 30 sec, 72°C 5 min finally 72°C 5 min on a TECHNE thermocycler (TC-3000, Bibby Scientific US, Burlington, NJ).

### Invasion Assay

Invasion of *P. gingivalis* into low-passage gingival fibroblast cells (GFC) was quantitated by an antibiotic protection assay, essentially as described previously [74] with minor modifications. Briefly, primary cultures of gingival cells were obtained from gingival explants and maintained in tissue culture. Gingival explants were obtained by consent (IRB: HSC-DB-10-0575). *P. gingivalis* cells at a multiplicity of infection (MOI) of 100 were added to a monolayer of GFC (10^6^ cells per well) in a 6-well culture plate, and incubated for 24 hours at 37°C in 5% CO_2_. External, non-adherent bacteria were removed by washing and remaining external bacteria were killed with the addition antibiotics for 1 h. After exposure to the antibiotics, the cells were washed and internal bacterial were released by lysis of the cells. Dilutions of the lysate were plated on BAP plates, cultured anaerobically, and finally, colony forming units (CFUs) of invasive bacteria were determined. Invasion was expressed as CFUs recovered after antibiotic treatment and fibroblast cell lysis.

### Auto-aggregation Assay

*P. gingivalis* cells, in the early to mid-log phase, were collected by centrifugation at 5,500×*g* for 5 min at RT, and resuspended in 10 ml of TSB to an optical density (OD_600_) of 2.0. Tubes were incubated at 4°C and the OD_600_ of each suspension was monitored for auto-aggregation every hour for 6 h. A 100 µl sample was taken from the top of each tube at 1 h intervals and monitored using an Eppendorf BioPhotometer (Eppendorf, Hamburg, Germany). Experiments were performed in triplicate. Slope was calculated using the formula rise/run, where ‘rise’ was the change in OD_600_ from time 0 to time 6 h and ‘run’ was 6 h.

### RNA Isolation

Cells of *P. gingivalis* were washed and resuspended in pre-reduced 1X phosphate buffered saline (PBS) to a final concentration of 1×10^8^ CFU/ml. Cells were recovered by centrifugation 5,500×g for 5 min and processed immediately for RNA extraction. Total RNA was isolated in triplicate. Bacterial cells were lysed using Trizol (Invitrogen, Life Technologies, Grand Island, NY) as described by the manufacturer for gram-negative bacteria. RNA was extracted with phenol-chloroform and precipitated with isopropanol. RNA preparations were washed with 70% ethanol, dissolved with RNase–free H_2_0 (Thermo Fisher Scientific, Houston, TX), and treated with DNase I for 1 h at 37°C (Fermentas, Life Sciences, Burlington, ON). The RNA was then purified on RNeasy columns (QIAGEN, Hilden, Germany). RNA concentration was estimated spectrophotometrically at A_260_.

### Real-Time Reverse Transcriptase PCR

Quantitative – real time- Reverse transcription Q-RT-PCR was performed with primers for the *P. gingivalis fimA* gene. Forward and reverse primers for *fimA* were designed for a target amplicon of 67 bp using a forward primer in nucleotide position 190310–190331, and a reverse primer in nucleotide position 190357–190376 (GenBank Accession no. NC_010729) (Table 2). Q-RT-PCR was performed on 100 ng total RNA in triplicate for all samples using the High Capacity cDNA RT Kit with SYBR Green Master Mix RT Kit (Applied Biosciences, Foster City, CA) according to the manufacturer’s protocol. The amplicon generation rate was determined by monitoring SYBR Green I fluorescence on a continuous basis. Fluorescence data were collected at a melting temperature greater than that of non-specific PCR products. A blank sample was included with RNase-free water in place of template. Amplification was done in a 96-well thin-wall plate (BIO-RAD) sealed with optical quality film using a protocol of: 10 min at 25°C and 120 min at 37°C for cDNA synthesis, 5 min at 85°C for reverse transcriptase inactivation, followed by 40 cycles of 95°C for 10 sec and 60°C for 30 sec for the data collection step. Melt curve analysis was performed using a protocol of: 1 min at 95°C, 1 min at 55°C, and increasing the temperature in 0.5°C increments starting at 55°C for 80 cycles of 10 sec each. The presence of a single PCR product was verified by a single melting temperature peak signifying a unique product. Data for *fimA* was converted into copy based on *fimA* standard curve using ATCC 33277 chromosomal DNA. Analysis software iCycler iQ version 3.1 optical software system (BIO-RAD).

### Invasion Microscope Observation

Gingival fibroblast cells (GFC) grown in Dulbecco’s Modified Eagle Medium (DMEM) plus fetal bovine serum (FBS) at 37°C in 5% CO_2_ were harvested by trypsin digest and added to CultureWell chambered coverglass 8-well slides (Grace Bio Labs, Bend, OR). GFC were allowed to attach overnight at 37°C in 5% CO_2_. Residual cells were removed by aspiration and 500 µl fresh DMEM plus FBS was added to each chamber well. Next, 1 ml of each *P. gingivalis* strain, grown to an early log phase, was pelleted by centrifugation at 5,500×g for 10 min and the supernatant was discarded. The cell pellets were washed with 1 ml of N18 buffer (10 mM glucose, 10 mM HEPES, 140 mM NaCl, 0.03 mM CaCl_2_, 2 mM MgCl_2_, 5 mM KCl, pH 7.5), pelleted as above, and resuspended in 1 ml of N18 buffer. One microliter of 4 mg/ml 5-(6) carboxyfluorescein -succinylester (fluorescein) stock (Invitrogen-Molecular Probes, Eugene, OR) was added to each resuspension and cells were incubated at 4°C for 30 min while covered with foil. Cells were pelleted, as above, and washed 3 times with 1 ml N18 buffer. Cells were resuspended in 500 µl of DMEM. *P. gingivalis* cells at a MOI of 50 were added to a monolayer of GFC (10^6^ GFC per well) in the 8-well chambered slides, and incubated for 24 hours at 37°C in 5% CO_2_. Following incubation, the DMEM bacterial suspension was removed from the chambered wells. GFC were washed one time with N18 buffer and cells were fixed by shaking with 4% formaldehyde for 30 min at room temperature. GFC were washed 2 times with N18 buffer. Next, 100 µl of rhodamine phalloidin (stock concentration 3.3 µM, Invitrogen-Molecular Probes) was added to each well, and plates were incubated for 30 min with gentle rocking at room temperature. Finally, an aliquot of DAPI (Invitrogen-Molecular Probes; final concentration 300 nM) was added to each well, and plates were incubated for an additional 2 minutes. The supernatant was aspirated, the slide chambers were removed, and the wells were mounted in PermaFluor hard-set mounting medium (Thermo Fisher Scientific). Invasion images were recorded at a 10x and 40x objective magnification using a Nikon Eclipse 80i epifluorescence microscope fitted with a Nikon DS-Qi1 digital camera, using the DAPI, fluorescein isothiocyanate (FITC), and Texas Red excitation/emission filter sets and Nikon NIS-Elements imaging software. Images were processed for contrast using ImageJ (Author: Wayne Rasband, National Institute of Mental Health, Bethesda, MD).

### Statistics

All experiments were repeated independently at least three times, with two or more replicates per experiment. Data comprised of two groups were analyzed for statistical significance using an unpaired *t* test, and data comprised of more than two groups were analyzed using one-way ANOVA and Tukey’s multiple comparison test. Statistical analysis was done with Excel software (Microsoft, Redmond, WA), and GraphPad Prism v6 (GraphPad Software, La Jolla, CA).
